# Overlapping Vitamin A Intervention Programs: Should We Be Concerned with Excessive Intakes?

**DOI:** 10.1093/jn/nxaa288

**Published:** 2020-10-06

**Authors:** Georg Lietz

**Affiliations:** Population Health Sciences Institute, Human Nutrition Research Centre, Newcastle University, Newcastle upon Tyne, United Kingdom

See corresponding article on page 2912.

## Vitamin A Intervention Programs and Their Effectiveness

Because ∼2.3% of all global deaths among children below the age of 5 y are attributable to vitamin A deficiency (VAD) annually (1), and provision of additional vitamin A (VA) reduces young child mortality by an average of 23%–30% (2, 3), programs have been initiated to reduce VAD through application of biannual high-dose VA supplements, micronutrient powder distribution, food fortification, or biofortification (4). Efforts to reduce VAD through these programs have resulted in an impressive 10% prevalence reduction worldwide from 39% in 1991 to 29% in 2013, yet VAD remains a global public health problem, especially in sub-Saharan Africa and South Asia (5). Where VAD is persisting, a combination of VA interventions will be required to achieve sustainable VA adequacy. However, overlapping VA interventions in the same population group have the potential of overexposing individuals at the high end of the intake distribution curve (6–8). This is particularly problematic if these interventions are not well coordinated between the different public and private sector entities that support these programs.

The article in this issue of *The Journal of Nutrition* by Sowa et al. (9) aimed to test the effect of VA program overlap by simulating the exposure of Zambian children to provitamin A biofortified foods and preformed VA food fortification using an animal model. Although it is not surprising that combined consumption of preformed VA with biofortified maize increased liver VA stores more effectively than either approach alone, their statement that this overlap resulted in “excessive” liver VA stores, despite downregulation of provitamin A conversion, potentially increases the concern about the safety of overlapping VA interventions.

## Contribution of Provitamin A Carotenoids to Excessive VA Stores?

Assuming that downregulation of provitamin A conversion through higher intakes of preformed VA is not protecting against excessive liver VA accumulation, the results by Sowa et al. (9) raise 2 important questions: *1*) can provitamin A carotenoids contribute to excessive liver VA stores when to date these nutrients have been regarded as “safe” owing to the downregulation of cleavage enzymes during high preformed VA intake (10, 11); and *2*) do we need to change the definition of the tolerable upper intake level (UL) for VA by including the contributions of provitamin A carotenoids?

Our current understanding of intestinal provitamin A carotenoid uptake and conversion assumes a saturable and protein-mediated uptake through a variety of intestinal transporters including Scavenger receptor class B type I (SCARB1), followed by the conversion into retinal via β-carotene 15,15′-oxygenase (BCO1) (11, 12). This process is regulated by the intestine-specific homeobox transcription factor (ISX), which enhances the expression of SCARB1 and BCO1 during VAD, but reduces their expression when sufficient dietary preformed VA is consumed (10, 11). Results from Sowa et al. (9) confirmed that increased intake of preformed retinol reduces the intestinal expression levels of *SCARB1* and *BCO1*, but did not confirm any involvement of *ISX* in gerbils. The lack of involvement of *ISX* in their study could be due to a mismatch of the amplicon sequence or a low qPCR amplicon efficiency, both of which were not tested. However, they did show that liver provitamin A carotenoid concentrations were higher in gerbils that were fed carotenoids and preformed VA than in those that only received provitamin A carotenoids, confirming a downregulation of provitamin A bioconversion by preformed VA. Contrarily, although Sowa et al. (9) confirm a downregulation of provitamin A bioconversion, they claim that constant exposure to both provitamin A and preformed VA would induce “excessive” VA liver stores compared with animals that were only fed similar amounts of preformed VA. In other words, the authors claim that the negative feedback mechanism exerted by preformed VA on provitamin A bioconversion does not prevent a potentially dangerous accumulation of VA in the liver. If this observation were true, the current definition of the UL for dietary VA would need to be revised, because it currently only applies to preformed retinol (13). Importantly, because the authors did not test a range of VA doses to establish hypervitaminosis or VA toxicity, this observation relies on using a liver VA cutoff of 1 μmol/g for “hypervitaminosis A.”

## What Are the Adequate Cutoff Values for “Hypervitaminosis A” or VA Toxicity?

It is important to note that the evidence for setting the cutoff for “hypervitaminosis A” is limited (14). The Biomarkers of Nutrition for Development Vitamin A Expert Panel agreed to set the cutoff for liver VA toxicity at 10 μmol/g liver, but used the term “hypervitaminosis A” for liver VA concentrations between 1 and 10 μmol/g liver until more data exist with regard to adverse effects (14). The use of these terminologies is potentially confusing, particularly as the older literature uses “hypervitaminosis A” for describing VA toxicity with clear pathophysiological indicators such as perisinusoidal fibrosis, hyperplasia, and hypertrophy of stellate cells (15–17). Most importantly, the lowest intake amount to cause pathophysiological confirmed chronic VA toxicity [200 μg/kg body weight (16)] is about 4 times higher than the UL for children aged 2–5 y of age [∼50 μg/kg body weight; based on the UL of 600 and 900 μg/d and a mean body weight of 12 and 18 kg for 2- and 5-y-old children, respectively (14, 18)]. “Hypervitaminosis A,” defined as liver VA concentrations >1 μmol/g liver (19, 20), has been reported to occur in >59% (21) or >64% (22) of children with VA intake amounts below the UL of 900 μg/d for this age group. Because the UL is defined as the “highest daily intake still considered to be safe for almost all healthy individuals in a specified group” (7), it raises the question of whether the liver VA cutoff for “hypervitaminosis A” is set too low. Thus, liver VA concentrations ∼1 μmol/g liver may simply describe “high” rather than “excessive” VA stores with no adverse health effects. Although Sowa et al. (9) refer to an observation of liver abnormality and bone fragility in rats at daily VA intake amounts below the UL for human adults [3000 μg/d (14)], the dose consumed in the cited animal study [2727 μg · rat^−1^ · d^−1^ (23); equivalent to 8536 μg/kg body weight] was 43 times higher than the lowest observed intake amount to cause chronic VA toxicity (200 μg/kg body weight) in humans (16). Furthermore, the same study also reported 7 times higher liver VA and 20 times higher serum retinyl ester concentrations in the high VA intake group than in the control group (23). It is important to remember that the current cutoff value for “hypervitaminosis A” (>1.05 μmol/g liver) was proposed by Olson in 1984 based on the hypothetical relation between liver and plasma concentrations of VA; i.e., that “once the liver storage mechanism for VA is largely saturated plasma levels of VA shall sharply rise” (19). Indeed, the theoretical cutoff for VA toxicity in liver of >1.05 μmol/g was subsequently accompanied by a cutoff value for plasma total VA concentration of >3.5 μmol/L to include circulating retinyl esters (20). Thus, according to Olson (20), “hypervitaminosis A” occurs when both criteria are confirmed, as described in rats suffering from liver abnormality and bone fragility (23). Consequently, owing to a lack of clear pathophysiological findings indicative of VA toxicity in animals or humans with liver concentrations ∼1 μmol/g liver, there is no sufficient evidence at this point in time to associate the current cutoff value with any adverse health effects. Furthermore, there is an urgent need to redefine the use of the terminology “hypervitaminosis A,” particularly in light of the older publications that use the same description for VA toxicity.

In conclusion, more data are needed to accurately determine cutoff values for biomarkers for assessing excessive VA status to enable a comprehensive evaluation of the safety of current ongoing and planned future VA interventions and programs. The study by Sowa et al. (9) raises an important question as to whether provitamin A carotenoid intake needs to be more carefully evaluated. However, because of the current uncertainty regarding the liver VA cutoff value for “hypervitaminosis A,” more research is urgently needed to establish a well-defined cutoff value for evaluating the safety of VA interventions and programs. A liver VA cutoff value could be applied to human populations by using the retinol isotope dilution method to estimate liver VA concentration indirectly in field settings.

## Notes

The author acknowledges funding from the Bill and Melinda Gates Foundation (OPP1115464).

Author disclosures: The author reports no conflicts of interest.

Abbreviations used: BCO1, β-carotene 15,15′-oxygenase; ISX, intestine-specific homeobox transcription factor; SCARB1, scavenger receptor class B type I; UL, tolerable upper intake level; VA, vitamin A; VAD, vitamin A deficiency.
